# A protein polymerization cascade mediates toxicity of non-pathological human huntingtin in yeast

**DOI:** 10.1038/srep18407

**Published:** 2015-12-17

**Authors:** Genrikh V. Serpionov, Alexander I. Alexandrov, Yuri N. Antonenko, Michael D. Ter-Avanesyan

**Affiliations:** 1 Bach Institute of Biochemistry, Research Center of Biotechnology of the Russian Academy of Sciences. 33, bld. 2 Leninsky Ave., Moscow 119071, Russia; 2 Belozersky Institute of Physico-Chemical Biology, Lomonosov Moscow State University, Leninskie gori, 1, bldg. 40, Moscow 119991, Russia

## Abstract

Several neurodegenerative amyloidoses, including Huntington disease, are caused by expansion of polyglutamine (polyQ) stretches in otherwise unrelated proteins. In a yeast model, an N-terminal fragment of mutant huntingtin with a stretch of 103 glutamine residues aggregates and causes toxicity, while its non-toxic wild type variant with a sequence of 25 glutamines (Htt25Q) does not aggregate. Here, we observed that non-toxic polymers of various proteins with glutamine-rich domains could seed polymerization of Htt25Q, which caused toxicity by seeding polymerization of the glutamine/asparagine-rich Sup35 protein thus depleting the soluble pools of this protein and its interacting partner, Sup45. Importantly, only polymers of Htt25Q, but not of the initial benign polymers, induced Sup35 polymerization, indicating an intermediary role of Htt25Q in cross-seeding Sup35 polymerization. These data provide a novel insight into interactions between amyloidogenic proteins and suggest a possible role for these interactions in the pathogenesis of Huntington and other polyQ diseases.

Mutations resulting in expansion of polyglutamine (polyQ) stretches in nine unrelated human proteins cause neurodegenerative diseases accompanied by deposition of amyloid protein aggregates formed by these proteins. The most frequent of these autosomal dominant disorders, Huntington disease, is caused by mutations that increase the number of CAG triplets in the first exon of the *HTT* gene coding for the huntingtin (Htt) protein. Individuals with up to 35 CAG repeats are healthy, but further expansion of polyQ stretch causes development of Huntington disease with a probability proportional to the repeat number^1^,^2^,^3^. Mutant Htt with an expanded N-terminal polyQ sequence aggregates and forms insoluble granular and fibrous deposits in affected neurons, mostly in the nucleus and, to a lesser extent, in the cytoplasm^4^,^5^,^6^. Despite extensive studies, the molecular bases of polyQ diseases are still unclear, though it was shown that the toxic effect of expanded polyQ proteins is related to interference with the normal function of various cellular proteins thus affecting different cellular processes. Indeed, pathological Htt impairs gene transcription, ubiquitin-proteasome system, causes mitochondrial dysfunction, dysregulation of Ca^2+^ homeostasis, impairment of axonal transport and genotoxic stress (for a review, see^7^). Experimental models, based on yeast *Saccharomyces cerevisiae*^8^, worm *Caenorhabditis elegans*^9^, fly *Drosophila melanogaster*^10^ and mouse *Mus musculus*^11^,^12^ have been established to elaborate the reasons of Htt toxicity on molecular and cellular levels.

As in humans, aggregation and toxicity of Htt in yeast increases with polyQ length and targeting of mutant Htt into the nucleus alters transcription of a subset of genes and decreases cell viability^13^. Cytoplasmically expressed Htt with an expanded polyQ region is also toxic, although its toxicity and aggregation depend on the presence of [*PSI*^+^] or [*PIN*^+^], the prion form of two glutamine/asparagine (Q/N)-rich proteins. The first is the translation termination factor Sup35, and the second is the Rnq1 protein with unknown function, which being in prion form facilitates the *de novo* appearance of other yeast prions including [*PSI*^+^]^8^. Besides, toxicity of Htt with expanded polyQ is modulated by the sequences flanking the polyQ stretch^14^ and by certain genome-encoded Q/N-rich proteins whose lack or overproduction can cause or abolish toxicity of mutant Htt^15^,^16^,^17^,^18^. Use of the yeast model has previously revealed that aggregation of mutant Htt results in defects of various processes, such as endocytosis, tryptophan metabolism, translation, cell cycle progression and endoplasmic reticulum-associated protein degradation^15^,^19^,^20^,^21^,^22^. The molecular bases of these defects are mostly unknown, though it was shown that an essential source of toxicity of Htt with elongated polyQ is related to its ability to induce polymerization and inactivation of the essential aggregation-prone Sup35 protein coupled with inactivation of another essential but not polymerizing protein, translation termination factor Sup45 via its sequestration into Sup35 aggregates^20^,^23^,^24^.

In yeast toxicity of mutant Htt is usually studied when this protein is expressed alone, however in humans the pathologic effects of the mutant *HTT* allele are manifested in the presence of its wild type counterpart, and some results indicate a role of non-pathological Htt in disease progression^25^. This role, however, can be complex, i.e. for Htt with shorter pathological polyQ, increasing the length of the polyQ in wild type Htt seems to exacerbate disease severity, while for mutant Htt with longer polyQ length, the effect is opposite^26^. The causes of these effects are not known, though there are several works that show wild type Htt to be included into mutant Htt aggregates^16^,^27^.

Earlier we used the yeast model to reveal sources of mutant Htt cytotoxicity^20^. In this work we continued modeling Huntington disease by assaying the role of wild type Htt. We observed that polymers of artificial proteins with long polyQ or polyQ interspersed with other residues, which are not toxic on their own^28^, seeded polymerization of wild type Htt, which resulted in cytotoxicity. Prion amyloids of the Rnq1 protein also induced polymerization and toxicity of wild type Htt. Remarkably, in both cases polymers of wild-type Htt could seed polymerization of Sup35 and other Q/N-rich proteins, which were not efficiently seeded by the initial amyloid templates. The obtained results may be relevant for elucidation of the pathogenesis of Huntington and other polyQ diseases as well as for understanding of importance of the phenomenon of amyloid interdependence.

## Results

### Htt25Q can form SDS-insoluble polymers and cause cytotoxicity

Htt with a polyQ stretch containing 103 glutamine residues (Htt103Q) fused to the green fluorescent protein (GFP) is frequently used to model Huntington disease in *S. cerevisiae*, because the aggregation-prone Htt103Q sequence strongly inhibits yeast growth thus mimicking toxicity, while GFP allows easy microscopic monitoring of Htt103Q-GFP aggregates. Importantly, the same protein but with a stretch of 25 glutamines, Htt25Q-GFP, does not form aggregates nor does it cause toxicity and, therefore, is usually used as a control. Earlier, it was shown that in yeast cells Htt103Q-GFP forms SDS-insoluble aggregates, which indicates their amyloid nature, since resistance to treatment with detergents is a common property of amyloids^29^,^30^. Amyloid nature of detergent-insoluble Htt103Q-GFP polymers formed in yeast cells is also supported by the fact that they contain generic amyloid epitopes for DNA aptamer binding^31^.

Here, we confirmed that, in contrast to Htt103Q-GFP, the Htt25Q-GFP protein did not exert toxicity in yeast and did not form fluorescent foci in their cells. However, SDD-AGE analysis allowed us to detect residual polymerization of Htt25Q-GFP seeded by the prion polymers of Rnq1 leading to an assumption that more efficient seeding of Htt25Q-GFP polymerization may be toxic. Since proteins with long polyQ form aggregates that sequester proteins with short polyQ, which cannot aggregate on their own^32^,^33^,^34^,^35^,^36^, one can suggest that polymers of proteins with long polyQ stretches act as efficient seeds for Htt25Q-GFP polymerization. Earlier we have constructed a set of polyQ/QX-Sup35MC fusion proteins by replacing the prion domain of Sup35 with either polyQ sequences of different length or polyQX sequences containing different number of QQQXQ repeats, where X represents any amino acid residue, and showed that such proteins can form non-toxic SDS-insoluble polymers in [*PIN*^+^] cells^28^,^37^. In this work we modified these proteins by replacing the Sup35C domain with either GFP or 2HA tags and studied their ability to induce Htt25Q-GFP polymerization and toxicity.

**Figure 1:**
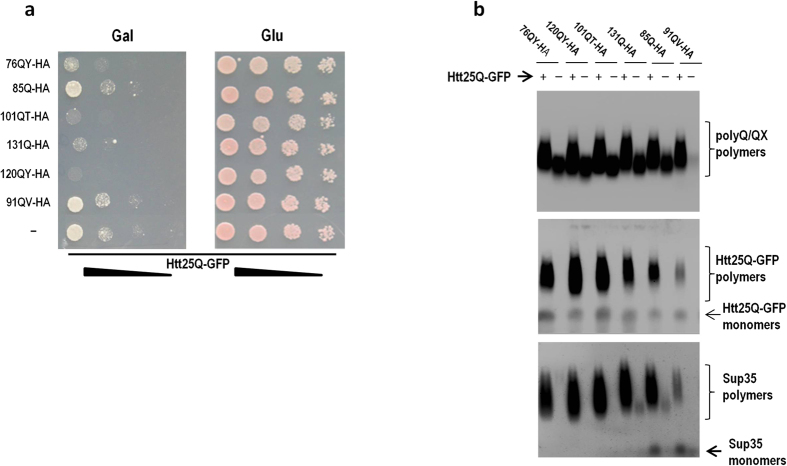
Toxicity of Htt25Q-GFP polymers correlates with their ability to seed Sup35 polymerization. (**a**) Induction of Htt25Q-GFP toxicity by co-production with polyQ/QX-HA proteins. The 74D-694 [*psi*^−^][*PIN*^+^] transformants each carrying a plasmid pair of which one plasmid expressed Htt25Q-GFP and another the indicated polyQ/QX-HA protein (-, an empty vector), were grown at 30^o^C in liquid SC-Ura-Leu medium with glucose, resuspended in the same medium but with raffinose instead of glucose and after 12 h incubation cell suspensions, diluted to an D600 of 1.0, were spotted onto SC-Ura-Leu plates with galactose as a sole carbon source (Gal) and incubated for 4 days. Equal spotting was controlled by parallel spotting the cells onto same selective plates containing glucose as carbon source (Glu). Four serial 5-fold dilutions of cell suspensions are shown. (**b**) PolyQ/QX-HA-dependent polymerization of Htt25Q-GFP and Sup35. 74-D694 [*psi*^−^][*PIN*^+^] transformants carrying plasmid pairs of which one plasmid either expressed Htt25Q-GFP (+) or did not express this protein (-) and another expressed the indicated polyQ/QX-HA protein, were grown as described in Methods. After incubation in SC-Ura-Leu Gal medium for 10 h, cells were harvested, lyzed and analyzed by SDD-AGE for the presence of SDS-insoluble polymers of polyQ/QX-HA, Htt25Q-GFP and Sup35. Anti-HA, -GFP and -Sup35C antibodies were used.

Simultaneous expression of Htt25Q-GFP with seven of the nineteen tested polyQ/QX-GFP proteins caused inhibition of growth of the 74-D694 [*psi*^*−*^][*PIN*^+^] strain (Fig. S1). In six representative proteins GFP was replaced with 2HA, which allowed us to study polymerization of the polyQX-HA and Htt25Q-GFP proteins separately. Notably, replacement of GFP with 2HA did not affect ability of these proteins to cause toxicity in the presence of Htt25Q-GFP (Fig. 1a). As expected, cells with polymers of polyQ/QX-HA proteins also contained Htt25Q-GFP polymers our suggestion that polyQ/QX polymers were efficient in seeding Htt25Q-GFP polymerization. Notably, expression of Htt25Q-GFP increased the size of polyQ/QX-HA polymers, suggesting that they grew via SDS-resistant binding of Htt25Q-GFP molecules (Fig. 1b).

### Htt25Q aggregates are detectable by fluorescence correlation spectroscopy

**Figure 2:**
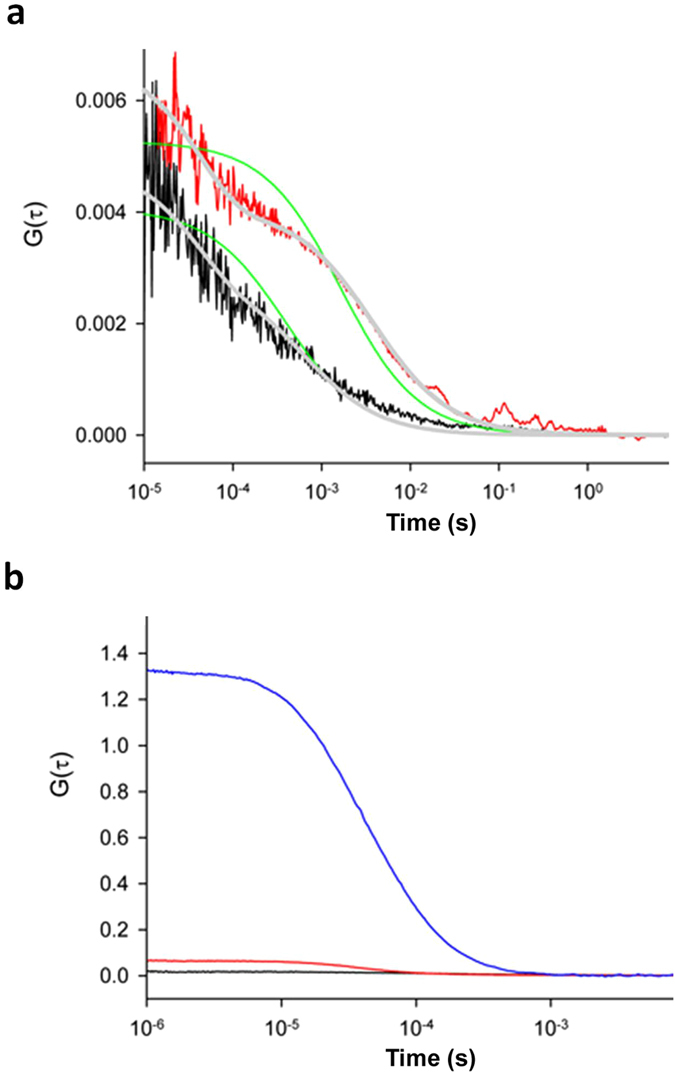
FCS analysis of HttQ25-RFP aggregation in lysates of cells co-expressing 120QY-HA. (**a**) Autocorrelation functions of HttQ25-RFP (black curve) and HttQ25-RFP+120QY-HA (red curve) samples. Green curves are best fit curves using equation (1) (See Methods) with the *τ*_*D*_ = 390 μs (HttQ25-RFP) and *τ*_*D*_ = 1600 μs (HttQ25-RFP+120QY-HA). Grey curves are best fit curves using equation (2) with the *τ*_*D*_ = 615 μs (HttQ25-RFP) and *τ*_*D*_ = 3740 μs (HttQ25-RFP+120QY-HA), and the *τ*_*R*_ = 39 μs (HttQ25-RFP) and *τ*_*R*_ = 43 μs (HttQ25-RFP+120QY-HA). (**b**) Autocorrelation functions of HttQ25-RFP (black curve), HttQ25-RFP+120QY-HA (red curve), and HttQ103-RFP (blue curve) samples measured under stirring conditions. HttQ103-RFP is shown for comparison.

The C-terminal moiety of the Htt25Q protein did not affect its ability to aggregate and cause toxicity, as was shown for Htt25Q fused with the red fluorescent protein (RFP) instead of GFP (Fig. S2 and S3). However, despite the presence of SDS-insoluble polymers, microscopic analysis of cells expressing Htt25Q fused with either GFP or RFP simultaneously with polyQ/QX proteins did not reveal visible fluorescent foci, which, for example, are typical for [*PIN*^+^] cells expressing Htt103Q-GFP (Fig. S4). We therefore exploited fluorescence correlation spectroscopy (FCS) to characterize aggregates of Htt25Q-RFP which this protein forms in the presence of 120QY-HA. FCS is a convenient tool for determining diffusion properties of fluorescent molecules both in cells and cell lysates^38^,^39^ and has been previously used to measure the size of the diffused Sup35-GFP aggregates in [*PSI*^+^] cells lacking fluorescent foci^40^. The autocorrelation function measured in the cell lysate containing both Htt25Q-RFP and 120QY-HA was shifted toward larger correlation times as compared with that of the lysate with Htt25Q-RFP alone, which reflects a dramatic decrease in the diffusion coefficient (*D*) of HttQ25-RFP upon its interaction with 120QY-HA (Fig. 2a). We also obtained autocorrelation functions *G(τ)* under continuous stirring conditions, which increased statistics of measurements of the parameter *N*, i.e. the number of particles (Fig. 2b). The amplitude of *G(τ)* at the limit of low *τ* is known to be inversely proportional to N^38^. This value for HttQ25-RFP was increased in the presence of 120QY-HA suggesting a considerable decrease in the number of particles N. These measurements enabled us to measure the average brightness of the particles by dividing the count rate by N. Namely, the particle brightness of HttQ25-RFP was on average ~4-fold lower than for that of HttQ25-RFP+120QY-HA suggesting a 4-fold average increase in the number of HttQ25-RFP molecules per particle. The autocorrelation function of HttQ103-RFP has bigger amplitude than in the case of HttQ25-RFP+120QY-HA, which is in line with the high degree of aggregation of the HttQ103-RFP protein.

### Toxicity of Htt25Q is related to depletion of soluble Sup35 and Sup45

In yeast a considerable source of Htt103Q-GFP cytotoxicity is depletion of a soluble form of the essential translation termination factor Sup35 due to seeding its polymerization^20^,^23^,^24^. We examined whether this was true for Htt25Q-GFP. SDD-AGE showed that polyQ/QX-HA polymers on their own were not efficient inducers of Sup35 polymerization (Fig. 1b). Only proteins with a uniform polyQ sequence, 85Q-HA and 131Q-HA, exhibited residual seeding activity, which agreed with the observation that polyQ seeded Sup35 polymerization better than polyQY^41^. In contrast, the presence of Htt25Q-GFP polymers always coincided with the appearance of Sup35 polymers. Remarkably, besides seeding Sup35 polymerization, polymers of wild type Htt could also serve as efficient templates for polymerization of other Q/N-rich proteins. C-terminal GFP fusions of Yir003w and Ylr278c, proteins, whose lack alleviates toxicity of HttQ103-GFP^15^, formed SDS-insoluble polymers in cells containing polymers of Htt25Q-RFP. Similarly to Sup35, polymerization of Yir003w and Ylr278c depended on polymers of Htt25Q-RFP, but not on the polymers of 120QY-GFP, which were used to induce Htt25Q-RFP polymerization (Fig. S2).

**Figure 3:**
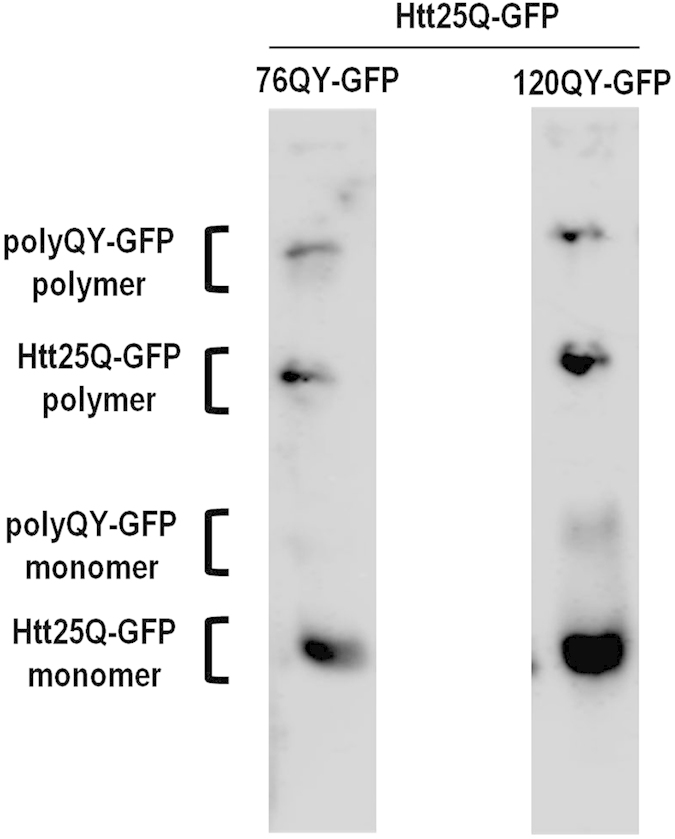
Comparison of the amount of Htt25Q-GFP and polyQY-GFP polymers. Transformants of the strain 74-D694 [*psi*^−^][*PIN*^+^] with plasmids expressing Htt25Q-GFP in combination with either 76QY-GFP or 120QY-GFP were grown as described in Methods. After incubation in SC-Ura-Leu Gal medium for 10 h, cells were harvested and cell lysates obtained. Then, cell lysates were loaded onto gels without boiling and run for half the length of the gel. The whole gel assembly was then boiled, and the electrophoretic separation was continued. The gels were blotted, and the blots were stained with anti-GFP antibody. Monomer, monomeric form of proteins; polymer, proteins derived from polymers after their dissolution by boiling. Abundances of polyQY-GFP and Htt25Q-GFP polymers were calculated after densitometry of blots using ImageJ software. Six independently-obtained transformants were analyzed and one of the images is presented. Relative polymer abundances were expressed as the means ± standard deviations. The amount of Htt25Q-GFP polymers exceeded that of 76QY-GFP and 120QY-GFP polymers by 1.4 ± 0.2 and 2.0 ± 0.3 fold, respectively.

An important question is why polymers of Htt25Q-GFP were much more efficient in seeding polymerization of Q/N-rich proteins than those of polyQ/QX-HA? One possibility is that Htt25Q-GFP was expressed under a control of much stronger promoter than polyQ/QX-HA, which resulted in a much larger amount of its polymers. However, quantitative analysis showed that amount of Htt25Q-GFP polymers exceeded that of 76QY- or 120QY-GFP polymers by no more than 2-fold (Fig. 3). Therefore, to explain efficient seeding of Sup35 polymerization by Htt25Q-GFP, it remains to suggest that the N-terminal sequence of Htt25Q-GFP (FLAG-epitope plus a sequence of seventeen amino acids encoded by the beginning of the first *HTT* exon) made this protein an efficient inducer of polymerization of Q/N-rich proteins.

**Figure 4:**
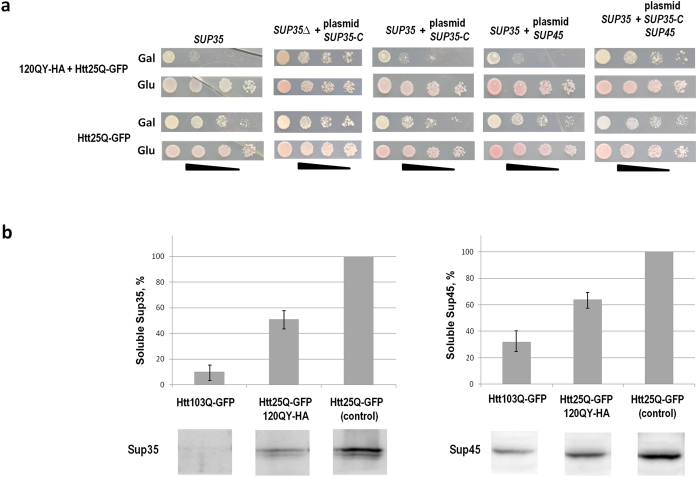
Co-production of 120QY-HA and Htt25Q-GFP causes toxicity which depends on depletion of soluble Sup35 and Sup45. (**a**) Growth of the 74-D694 [*psi*^−^][*PIN*^+^] derivatives expressing either 120QY-HA and Htt25Q-GFP, or Htt25Q-GFP alone and carrying plasmids with *SUP35*, *SUP35-C* and *SUP45*, as indicated above the panels. *SUP35*, the strain with chromosomal wild type *SUP35*; *SUP35*∆+plasmid *SUP35-C*, the strain disrupted for *SUP35*, which carries centromeric plasmid with *SUP35-C* encoding Sup35 devoid of the prionogenic NM region; *SUP35*+plasmid *SUP35-C*, the strain with chromosomal wild type *SUP35* which carries centromeric plasmid with *SUP35-C*; *SUP35*+plasmid *SUP45*, the strain with chromosomal wild type *SUP35*, which carries centromeric plasmid with *SUP45*; *SUP35*+plasmid *SUP35-C SUP45*, the strain with chromosomal wild type *SUP35*, which carries centromeric plasmid with *SUP35-C* and *SUP45*. Four serial 5-fold dilutions of cell suspensions are shown. For other details, see legend to Fig. 1. (**b**) Centrifugation analysis of levels of soluble Sup35 and Sup45. Cell lysates were fractionated by ultracentrifugation and supernatants were analyzed by SDS-PAGE and Western blotting. Staining with either anti-Sup35NM (Sup35) or anti-Sup45 (Sup45) antibodies. Relative abundances of soluble Sup35 and Sup45 in transformants expressing HttQ103-GFP, HttQ25-GFP in combination with 120QY-HA or non-toxic and non-aggregating HttQ25-GFP alone, used as a control, were calculated after densitometry of blots using ImageJ software. Three independently-obtained transformants of each type were analyzed and the relative protein abundances presented on histograms are expressed as the means ± standard deviations. A typical blot image is presented.

The obtained data showed that Htt25Q-GFP polymers induced polymerization of Sup35, which might be the cause of cytotoxicity. In agreement with this, we observed that similarly to Htt103Q-GFP, expression of the non-aggregating form of Sup35, which lacked the NM region (Sup35C), instead of the full-length protein, alleviated the toxic effect of HttQ25-GFP, which this protein manifested in the presence of 120QY-HA (Fig. 4a). Importantly, Sup35C co-expressed with wild type Sup35 did not improve growth of cells which co-produced Htt25Q-GFP and 120QY-HA, suggesting that not only depletion of soluble Sup35, but also appearance of its polymers contributed to Htt25Q-GFP toxicity. This corresponded to the results obtained for Htt103Q-GFP, toxicity of which depends not only on depletion of soluble Sup35, but also on appearance of its polymers, which is accompanied by sequestration of Sup45 through its binding to these polymers^20^. Indeed, similarly to Htt103Q-GFP, polymers of Htt25Q-GFP caused depletion of soluble Sup35 and Sup45, though to a lesser extent (Fig. 4b). However, in contrast to Htt103Q-GFP, overproduction of Sup45 alone did not reduce Htt25Q-GFP toxicity and only simultaneous overproduction of Sup35C and Sup45 in the strain with the chromosomal wild type *SUP35* gene partially abrogated the toxic effect of Htt25Q-GFP in cells co-producing 120QY-HA (Fig. 4a).

**Figure 5:**
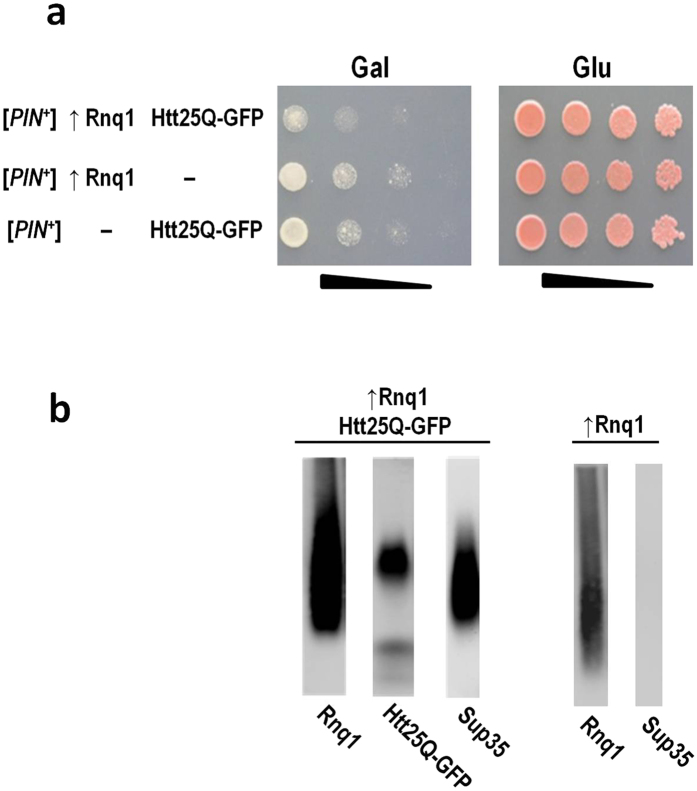
Htt25Q-GFP aggregates and causes toxicity in [*PIN*^+^] cells overexpressing Rnq1. (**a**) Growth of the 74-D694 [*psi*^−^][*PIN*^+^] strain which carries multicopy plasmids encoding either Rnq1 or Htt25Q-GFP, or both proteins simultaneously, was analyzed as described in legend to Fig. 1. Four serial 5-fold dilutions of cell suspensions are shown. (**b**) Polymerization of Sup35 depends on polymers of Htt25Q-GFP generated in [*PIN*^+^] cells upon overproduction Rnq1. Polymers of Rnq1, Htt25Q-GFP and Sup35 proteins in lysate of 74-D694 [*psi*^−^][*PIN*^+^] were visualized by SDD-AGE followed by Western blotting with the use anti-GFP, anti-Rnq1 and anti-Sup35 polyclonal antibodies.

As was mentioned above, [*PIN*^+^] promoted residual Htt25Q-GFP polymerization, which did not result in cytotoxicity. Inefficient seeding of Htt25Q-GFP polymerization could be due to low levels of Rnq1 polymers in [*PIN*^+^] cells which is supported by the observation that [*PIN*^+^] Stimulates polymerization of Pub1, a yeast protein with Q/N-rich domains, only at increased levels of both Rnq1 and Pub1^41^. Indeed, Htt25Q-GFP polymerized and manifested toxicity in [*PIN*^+^] cells overproducing Rnq1. Importantly, as in other studied cases, polymerization of Htt25Q-GFP was accompanied by the appearance of Sup35 polymers (Fig. 5). However, these polymers were not observed in [*PIN*^+^] cells overproducing Rnq1 in the absence of Htt25Q-GFP, which confirmed that Sup35 polymerization depended exclusively on Htt25Q-GFP.

### Soluble Htt25Q can manifest toxicity under non-optimal growth conditions

**Figure 6:**
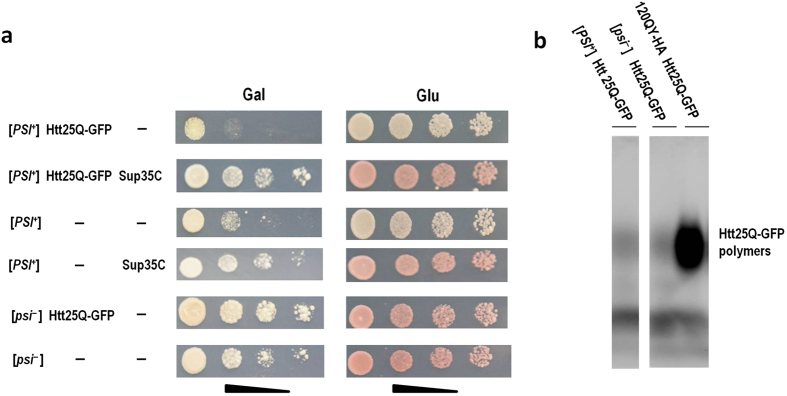
Toxicity of Htt25Q-GFP in [*PSI*^+^] cells is not related to its polymerization. (**a**) Growth of the 74-D694 [*PSI*^+^][*PIN*^+^] strain and its [*psi*^−^][*PIN*^+^] derivative carrying either a multicopy plasmid encoding Htt25Q-GFP or empty vector (−). Where indicated, transformants in addition carry either centromeric plasmid encoding Sup35C, or empty vector (−). Growth of transformants was analyzed as described in legend to Fig. 1. Four serial 5-fold dilutions of cell suspensions are shown. (**b**) Htt25Q-GFP does not form polymers both in [*PSI*^+^][*PIN*^+^] in [*psi*^−^][*PIN*^+^] cells of 74-D694. The transformant simultaneously expressing Htt25Q-GFP and 120QY-GFP was used as a positive control for polymer formation. Polymers of Htt25Q-GFP were visualized by SDD-AGE followed by Western blotting and staining with anti-GFP antibody.

Not only [*PIN*^+^], but also [*PSI*^+^] provokes toxicity of Htt103Q-GFP^42^. This prompted us to examine the ability of [*PSI*^+^] to cause Htt25Q-GFP toxicity and polymerization. As expected, overproduction of Htt25Q-GFP inhibited growth of [*PSI*^+^] cells and this effect disappeared upon expression of non-aggregating Sup35C (Fig. 6a). Surprisingly, Htt25Q-GFP did not polymerize in [*PSI*^+^] cells (Fig. 6b), though this protein formed polymers in [*PIN*^+^] cells overproducing Rnq1. Therefore, the observed toxicity of Htt25Q-GFP in [*PSI*^+^] cells was not related to its polymerization. To explain the reasons for growth inhibition, we proposed that Htt25Q-GFP strengthened [*PSI*^+^] to a level, which was incompatible with cell viability, thus resembling so called suicidal [*PSI*^+^]^43^. However, this was not the case, since expression of Htt25Q-GFP in [*PSI*^+^] cells did not significantly reduce the level of soluble Sup35 or increase the amount of its polymers (Fig. S5). Importantly, [*PSI*^+^] cells grew slower than [*psi*^−^] on medium with galactose instead of glucose, which was used to induce Htt25Q-GFP expression (Fig. 6a), and the effect of growth inhibition became more pronounced upon production of Htt25Q-GFP. Taking this into consideration, we hypothesized that soluble Htt25Q-GFP exerts a cryptic toxic effect, which can be observed only at non-optimal conditions for yeast growth. This possibility was supported by the observation that non-polymerized Htt25Q-GFP also manifested toxicity in yeast cells incubated in stressful environments which inhibited their growth but did not stimulate Htt25Q polymerization (Fig. S6). Interestingly, at optimal growth conditions expression of Htt25Q-GFP slightly increased the growth rate of [*psi*^−^] cells (Fig. 6 and S6).

## Discussion

Earlier we have reported that both the amount of Htt103Q polymers and their toxicity increase in the presence of the [*PIN*^+^] prion, while the protein with a shorter polyQ stretch, Htt25Q, does not aggregate nor manifest toxicity in these cells^20^. However, here we observe that Htt25Q forms trace amounts of polymers in [*PIN*^+^] cells, though this is not accompanied by any signs of toxicity. This prompted us to perform a search for conditions promoting polymerization of Htt25Q.

### Molecular basis of Htt25Q cytotoxicity

Expression of several aggregation-prone polyQ/QX-containing proteins, which we constructed earlier^28^, induces aggregation of Htt25Q as was shown both by detecting its SDS-insoluble polymers and measuring mobility of Htt25Q-RFP molecules using FCS. Importantly, analyses of HttQ25 aggregation suggest that this protein cannot polymerize on its own and its polymers arise via constant seeding on polyQ/QX templates, which agrees with a small number of Htt25Q monomers per aggregate shown by FCS. It is also noteworthy that since all tested polyQ/QX polymers do not cause overt toxicity on their own and do not induce efficient Sup35 polymerization, the observed toxicity and Sup35 polymerization seems to be mediated by Htt25Q. As we and others have shown earlier, toxicity of Htt103Q is due to both depletion of soluble Sup35 and appearance of its polymers which bind and sequester another essential protein, Sup45^20^,^23^,^24^. The data obtained in this work show that the mechanisms of Htt25Q toxicity are generally the same, because toxicity can be notably ameliorated by simultaneous overproduction of Sup45 and the non-polymerizing form of Sup35. Similarly to polyQ/QX proteins, the [*PIN*^+^] prion also facilitates Htt25Q polymerization and toxicity, but only upon overproduction of Rnq1. Toxicity of Htt25Q in this case is, most probably, also related to Sup35 polymerization and, remarkably, Sup35 polymerization depends on polymers of Htt25Q but not those of Rnq1. Therefore, in all studied cases, efficient polymerization of Sup35 accompanied with toxicity is induced by Htt25Q polymers, but not by polymers that seed Htt25Q polymerization. In contrast to Rnq1, prion amyloids of Sup35 do not seed polymerization of Htt25Q, thus representing an example of a seeding asymmetry, since polymers of Htt25Q are able to seed Sup35 polymerization. Further analysis has shown that the soluble form of Htt25Q exerts a cytotoxic effect which can be observed only upon inhibition of cell growth by stressful environments. It is however noteworthy that the toxic effect of soluble Htt25Q may also contribute to inhibition of cell growth upon co-expression of Htt25Q and polyQ/QX, because only a small portion of Htt25Q is aggregated in such cells.

### Intermediary cross-seeding and protein polymerization cascades

**Figure 7:**
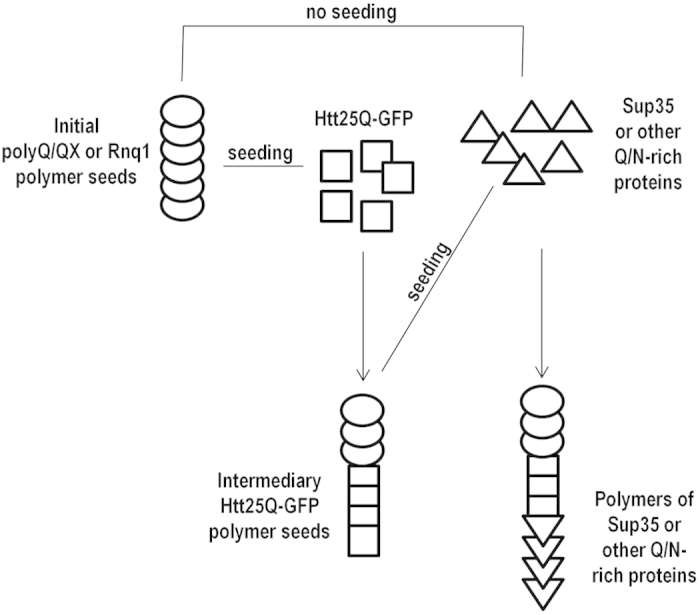
Intermediary cross-seeding and a protein polymerization cascade.

The data obtained in this work provide a new insight into the phenomenon of amyloid interdependence. Earlier it was demonstrated that polymers formed by proteins with polyQ or Q/N-rich domains, can seed polymerization of multiple other proteins with similar domains 20,41,42,44, which can influence the toxicity of polyQ amyloids^20^,^42^,^47^,^48^. This means that conversion of a single polyQ or Q/N-rich protein into a polymer may cause conversion of other proteins of this class into a polymeric state. Notably, such polymers can appear due to cross-seeding by the same initial polymer seed, or, alternatively, the process of cross-seeding can be sequential, representing a cross-polymerization cascade, which forms a network of interdependent polymers. The latter possibility, which can be described as intermediary seeding, is in agreement with our results showing that Htt25Q polymers, which are seeded by polyQ/QX or Rnq1 amyloids, can induce polymerization of other Q/N-rich proteins which do not aggregate in the presence of the initial seeding amyloids. (Fig. 7). Intermediary polymerization seeding shown for Htt25Q is likely to be widespread and other proteins with such properties remain to be found.

### Possible implications for polyQ disorders

We show that in yeast polymerization and toxicity of wild type Htt can be induced by other proteins with long polyQ/QX sequences. This allows us to hypothesize that in humans polymerization and toxicity of disease-associated proteins with non-pathological polyQ can be induced by other proteins with elongated Q/N-rich regions, even by those whose association with diseases has not been shown earlier. In addition, our data on the existence of protein polymerization cascades suggest that such a mechanism may be involved in the pathogenesis of amyloid diseases, especially of those which involve aggregation of Q/N-rich proteins. Further research is needed to assess whether our observations are indeed relevant to emergence and manifestation of neurological symptoms.

## Methods

### Plasmids, strains and growth conditions

The plasmids used in this study are described in Table 1. The plasmids encoding polyQX-GFP (where X is any amino acid residue) were constructed from the respective polyQX-Sup35MC-encoding plasmids of the pSBSE-QX series^28^,^37^ using *in vivo* recombination in yeast. YEp181-SUP35NM-GFP^49^ was digested by *Mlu*I and *Mls*I, mixed with a *Pvu*II- and *Hpa*I-digested pSBSE-QX and this mixture was introduced into yeast cells by the standard transformation protocol^50^. Then, plasmids were isolated from yeast cells, used to transform *E. coli*, and after extraction, were verified by sequencing. The YEp181-QX-HA plasmids encoding proteins with polyQX-2HA (2HA, double hemagglutinin tag) were generated by ligating the *Mlu*I-*Nar*I fragment of pSBSE-QX^28^,^37^, *Nar*I-*Bam*HI fragment of YCp111-Sup35NM-2HA^41^ and the *Bam*HI-*Mlu*I fragment of YEplac-181-76QY-GFP. The plasmid simultaneously encoding 120QY-2HA and Htt25Q-GFP was also obtained by ligating three fragments. The first fragment was obtained from YEp181-120QY-2HA by *Pvu*I digestion, subsequent Klenow fragment treatment, and then digestion by *Mlu*I. The Htt25Q-GFP-encoding fragment was obtained by treating the plasmid p25Q-GFP with by *Age*I and *Mlu*I and the vector fragment was obtained from pYES2 by treatment with *Age*I/*Pvu*II. The pRS315-SUP35-C+SUP45 plasmid containing the *SUP35C* and *SUP45* genes was constructed by introducing the *Pvu*II-*Xba*I fragment of the plasmid pRS315-SUP35-C^51^ encoding *SUP35-C* into the *Pvu*II-*Xba*I sites of the pRS315-SUP45 plasmid^52^.

The following strains were used: 74-D694 *MAT**a** ura3-52 leu2-3,112 trp1-289 his3*-Δ*200 ade1-14* [*psi*^−^][*PIN*^+^]^53^ and its derivative 74-D694ΔS35, in which *SUP35* was disrupted by insertion of *TRP1* with the use of pSUP35::TRP1 plasmid^51^, as well as W303 *MAT**a** ade2-1 trp1-1 leu2-3,112 his3-11,15 ura3-52 can1-100* [*psi*^−^][*PIN*^+^]^54^. Yeast cells were grown at 30 °C on plates with rich (1% yeast extract, 2% peptone, 2% glucose) or synthetic (0.67% yeast nitrogen base, 2% glucose and required amino acids) media. To induce the synthesis of Htt25Q-/103Q-GFP or -RFP chimeric proteins, transformants with corresponding plasmids were incubated in liquid selective media with 2% raffinose as a sole carbon source to mid-log phase and then transferred to media with 2% galactose instead of raffinose and cells were grown for 10 h. For induction of Htt25Q-/103Q-GFP or -RFP on solid media, cell suspensions incubated in raffinose-containing media were spotted onto plates containing selective media with 2% galactose as a sole carbon source. All growth assays were done in triplicate.

Cells for fluorescent microscopy were grown as described above, except that the growth medium contained 2-fold excess of adenine (40 mg/ml) in order to prevent the accumulation of fluorescent red pigment which is characteristic of *ade1* and *ade2* mutants. Cells were photographed using a Zeiss Axioskop fluorescent microscope and Olympus cooled CCD camera.

### Preparation of yeast cell lysates

Yeast cultures grown in liquid selective media were harvested, washed in water and lyzed by beating with glass beads in buffer A: 30 mM Tris-HCl, pH 7.4, 150 mM NaCl, 1 mM dithiothreitol and 1% Triton X-100. To prevent proteolytic degradation, the buffer was supplemented with 10 mM phenylmethylsulfonyl fluoride and Complete^TM^ protease inhibitor cocktail (Roche Applied Science). Cell debris was removed by centrifugation at 1500 g for 4 min.

### Centrifugation

To compare amount of soluble and aggregated Sup35 and Sup45 200 μl of yeast cell lysates were centrifuged at 100 000 g (Beckman Optima MAX-TL ultracentrifuge) for 1 h at 4 °C.

### Electrophoresis and blotting

SDS-PAGE was performed according to the standard protocol in 10% polyacrylamide gels and SDD-AGE according to^29^ with some modifications^28^. Protein loads were equalized for each gel. To compare amount of Htt25Q-GFP and 120QY-GFP in the aggregated and soluble fraction in a single gel, the standard SDS-PAGE procedure was modified as described^55^. Briefly, yeast cell lysates were mixed with sample buffer, incubated for 1 min at room temperature, loaded onto the gel and electrophoresis was run for ~40 min. Monomers entered the gel, whereas polymers were trapped at the start of the stacking gel. To dissolve and analyze the polymers, sample buffer was loaded into the wells, the gel was placed in a tall, unsealed polystyrene bag and boiled for 5 min with the top of the bag above the surface of the boiling water. After that the wells were filled with sample buffer again and the separation was continued. Proteins were transferred from gels to nitrocellulose membranes sheets (Macherey-Nagel) by 5 h vacuum-assisted capillary blotting (agarose gels), or using the Biorad electroblotting system (polyacrylamide gels). Rabbit polyclonal antibodies against GFP (Santa-Cruz), Sup35NM or Sup35C fragments, Sup45 and murine monoclonal antibodies against HA (Sigma) were used. Bound antibody was detected using the ECL West Dura substrate (Thermo Scientific). All electrophoresis experiments were repeated at least two times.

### Fluorescence correlation spectroscopy

FCS measurements were performed on yeast cell lysates diluted 100-fold with 25 mM Tris buffer (pH = 7.4). The home-made FCS experimental setup was described previously^56^. Briefly, excitation of fluorescence and detection utilized a Nd:YAG solid state laser with a 532-nm beam attached to an Olympus IMT-2 epifluorescent inverted microscope equipped with a 40×, NA 1.2 water immersion objective (Carl Zeiss). The fluorescence light passed through an appropriate dichroic beam splitter and a long-pass filter and was imaged onto a 50-μm core fiber coupled to an avalanche photodiode (SPCM-AQR-13-FC, PerkinElmer Optoelectronics, Vaudreuil). The signal from an output was sent to a PC using a fast interface card (Flex02-01D/C, Correlator.com, Bridgewater). The data acquisition time was 30 s. The fluorescence was recorded from the confocal volume located at about 50 μm above the coverslip surface with 50 μl of the buffer solution added. Some of the data were collected under the conditions of stirring a suspension by a paddle-shaped 3-mm plastic bar rotated at 600 rpm. To calibrate the setup, we recorded the autocorrelation function of fluorescence of a solution of Rhodamine 6G (Fluka) which was characterized by the correlation time *τ*_*D*_ estimated from the equation (1). Assuming the diffusion coefficient of the dye to be 2.5 × 10^−6^ cm^2^/s, the value of the confocal radius ω = 0.42 μm was obtained. The correlated fluorescence emission signals were fitted to the three-dimensional autocorrelation function^57^.


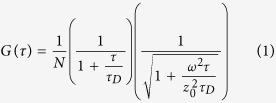


with τ_D_ being the characteristic correlation time during which a molecule resides in the observation volume of radius ω and length z_0_, given by τ_D_ = ω^2^/4*D*, where *D* is the diffusion coefficient, N is the mean number of fluorescent particles in the confocal volume, N = 1/G(τ à 0).

It has been shown that the description of G(τ) for GFP type of proteins requires consideration of proton-transfer reactions in the protein chromophore center leading to additional fluctuations of the fluorescent signal^58^, namely:


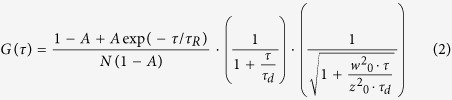


where *τ*_*R*_ is a characteristic time of the fluctuations and A is a fraction factor.

## Additional Information

**How to cite this article**: Serpionov, G. V. *et al*. A protein polymerization cascade mediates toxicity of non-pathological human huntingtin in yeast. *Sci. Rep*. **5**, 18407; doi: 10.1038/srep18407 (2015).

## Supplementary Material

Supplementary Information

## Figures and Tables

**Table 1:** Plasmids.

Plasmid	Characteristics	Reference
p25Q-GFP/RFP	Multicopy *URA3* pYES2 plasmid, encoding fusion of Htt25Q with GFP/RFP under the control of *GAL1* promoter	8
p103Q-GFP/RFP	Multicopy *URA3* pYES2 plasmid, encoding fusion of Htt103Q with GFP/RFP under the control of *GAL1* promoter	8
YEp181-QX-GFP	Multicopy *LEU2* plasmid, encoding polyQ/QX stretches of different length C-terminally fused with GFP	28
YEp181-QX-2HA	The same as plasmids of the QX-GFP series encoding 85Q, 76QY, 101QT, 131Q, 120QY or 91QV but with 2HA instead of GFP	This work
pYES2-120QY-2HA+Htt25-GFP	Multicopy *URA3* pYES2 plasmid, co-expressing under the control of *GAL1* promoter fusion of Htt25Q with GFP and 120QY-2HA	This work
pRS315-SUP35-C+SUP45	Centromeric *LEU2* pRS315 plasmid, encoding simultaneously the C-domain of Sup35 (Sup35C) and Sup45	This work
pRS315-SUP35-C	Centromeric *LEU2* pRS315 plasmid encoding the Sup35C protein	51
pRS315-SUP45	Centromeric *LEU2* pRS315 plasmid encoding the Sup45 protein	52
pALX1	Integrative vector with the *prb1*::*LEU2* disruption allele	59
YEp181	Multicopy *LEU2* plasmid	60
pYES2	Multicopy *URA3* plasmid	Invitrogen
pRS315	Centromeric *LEU2* plasmid	61
YEplac181-RNQ1	Multicopy *LEU2* plasmid, encoding Rnq1	41
pSUP35::TRP1	Integrative vector with the *sup35::TRP1* disruption allele	51
