# Supplementary material for: A protein polymerization cascade mediates toxicity of non-pathological human huntingtin in yeast
Source: Sci Rep. 2015 Dec 17;5:18407. doi: 10.1038/srep18407 (PMC4682096; doi:10.1038/srep18407)
Supplement: Supplementary Information [file srep18407-s1.pdf]

A protein polymerization cascade mediates toxicity of non-pathological human huntingtin in yeast

Genrikh V. Serpionov, Alexander I. Alexandrov, Yuri N. Antonenko & Michael D. Ter-Avanesyan

Supplementary material

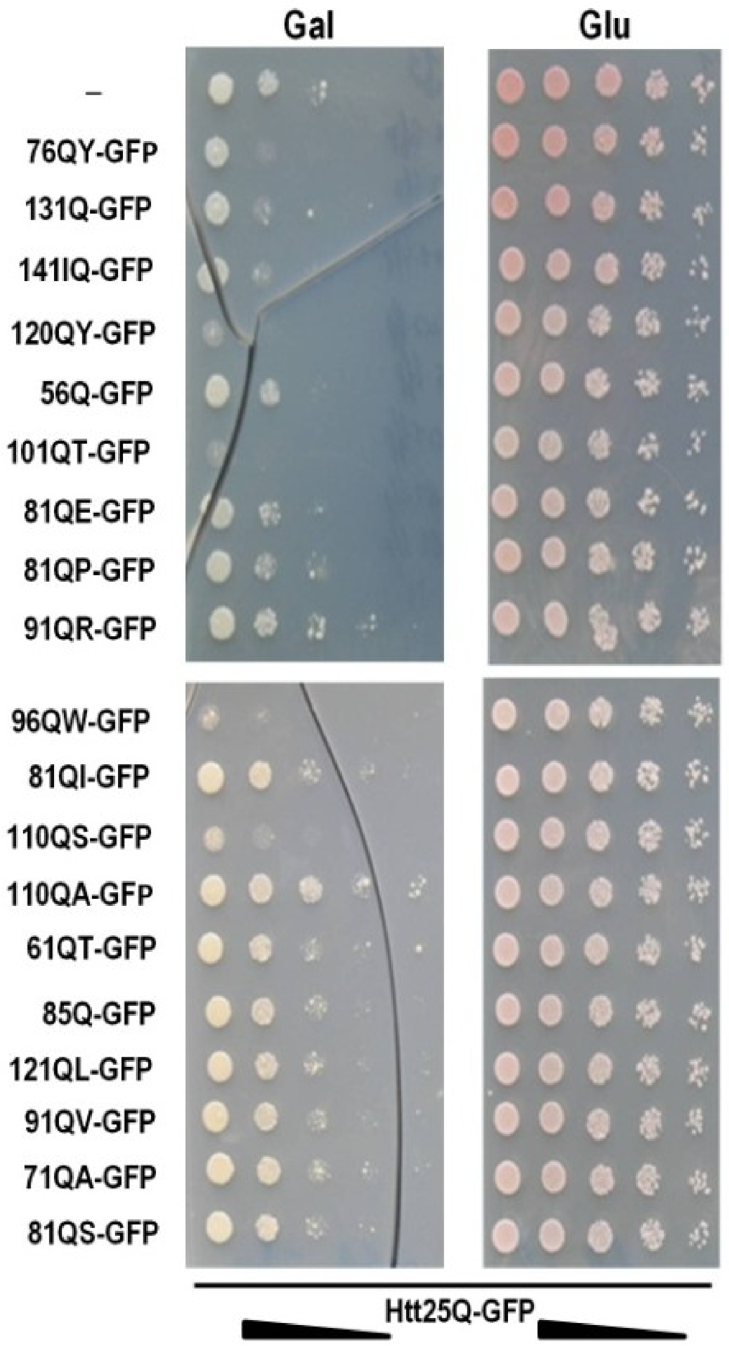

Figure S1 | Toxicity of HttQ25-GFP can be induced by co-production of polyQX-GFP proteins. The growth of [*psi*$^{-}$][*PIN*$^{+}$] transformants of the strain 74-D694 each carrying a plasmid pair of which one plasmid expresses Htt25Q-GFP and another the indicated polyQ/QX-GFP protein (-, an empty vector) was analyzed as described in legend to Fig. 1. Five serial 5-fold dilutions of cell suspensions are shown. Co-production of HttQ25-GFP with either one of the 76QY-, 131Q-, 141Q-, 120QY-, 101QT-, 96QW- or 110QS-GFP proteins causes an overt inhibition of growth.

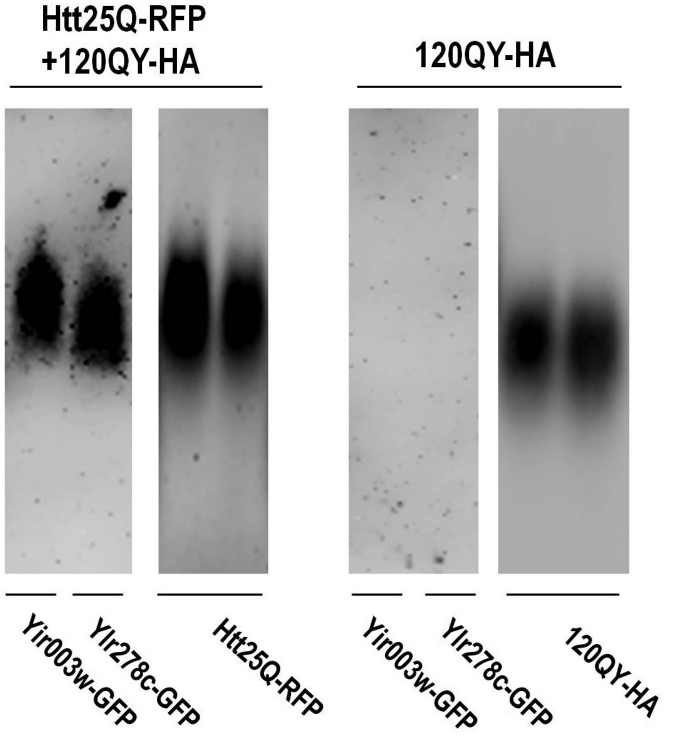

Figure S2 | 120QY-HA stimulates Htt25Q-RFP aggregation and induces aggregation of the Q/N-rich proteins Yir003w and Ylr278c. Yir003w and Ylr278c form SDS-insoluble polymers in the presence of Htt25Q-RFP aggregates. Polymers of Htt25Q-RFP, Yir003w-GFP and Ylr278c-GFP visualized by SDD-AGE followed by Western blotting with antibodies against FLAG (Htt25Q-RFP), HA (120QY-HA) and GFP (Yir003w-GFP or Ylr278c-GFP).

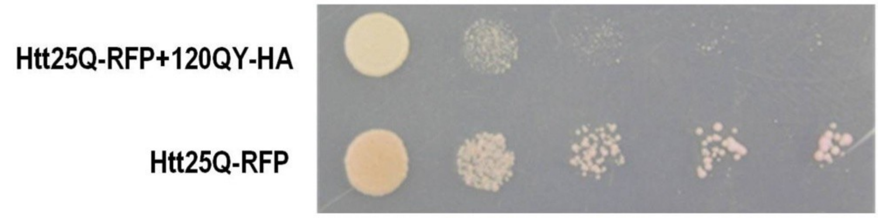

Figure S3 | 120QY-HA stimulates Htt25Q-RFP toxicity. Growth of the 74-D694 [*psi*$^-$][*PIN*$^+$] strain carrying plasmids encoding either Htt25Q-GFP or Htt25Q-GFP in combination with 120QY-HA was analyzed as described in legend to Fig. 1. Five serial 5-fold dilutions of cell suspensions are shown.

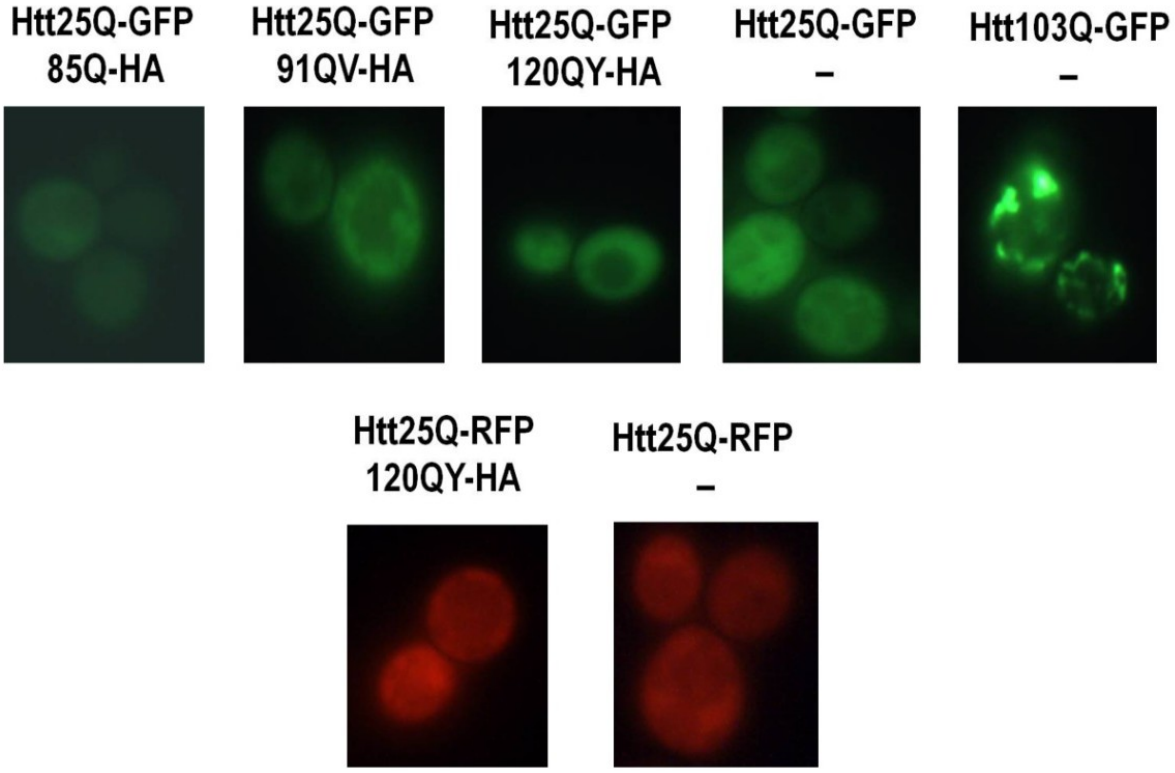

Figure S4 | Htt25Q-GFP, unlike HttQ103-GFP, does not form fluorescently-distinguishable foci, despite its aggregated state in the presence of polyQX-HA. Transformants contain pairs of plasmids encoding either Htt25Q-GFP or Htt25Q-RFP and the indicated polyQ/QX-HA protein. (-) designates an empty vector. Transformants expressing only Htt25Q-GFP or Htt103Q-GFP were used as controls.

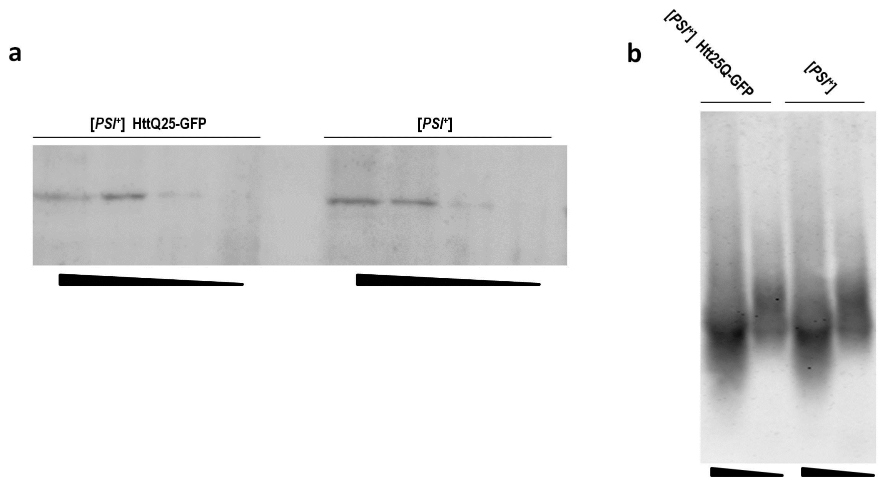

Figure S5 | Expression of Htt25Q-GFP in [*PSI*$^+$] cells does not affect the levels of Sup35 monomers and polymers. Cells of the strain 74-D694 [*PSI*$^+$][*PIN*$^+$] carrying either multicopy Htt25Q-GFP plasmid or empty vector were grown as described in legend to Fig. 1. (**a**) The levels of monomeric Sup35, SDS-PAGE analysis. Serial 3-fold dilutions of samples are shown. The samples were not boiled before loading onto the gel, which allowed monomeric Sup35 to enter the gel[55]. To reduce proteolytic degradation of soluble Sup35 74-D694 [*PSI*$^+$][*PIN*$^+$] was disrupted for the vacuolar proteinase B-encoding *PRB1* gene[28,59], (**b**) The levels of polymer Sup35, SDD-AGE analysis, 1:3 and 1:9 dilutions of samples are shown. Blots were stained with anti-Sup35 antibody.

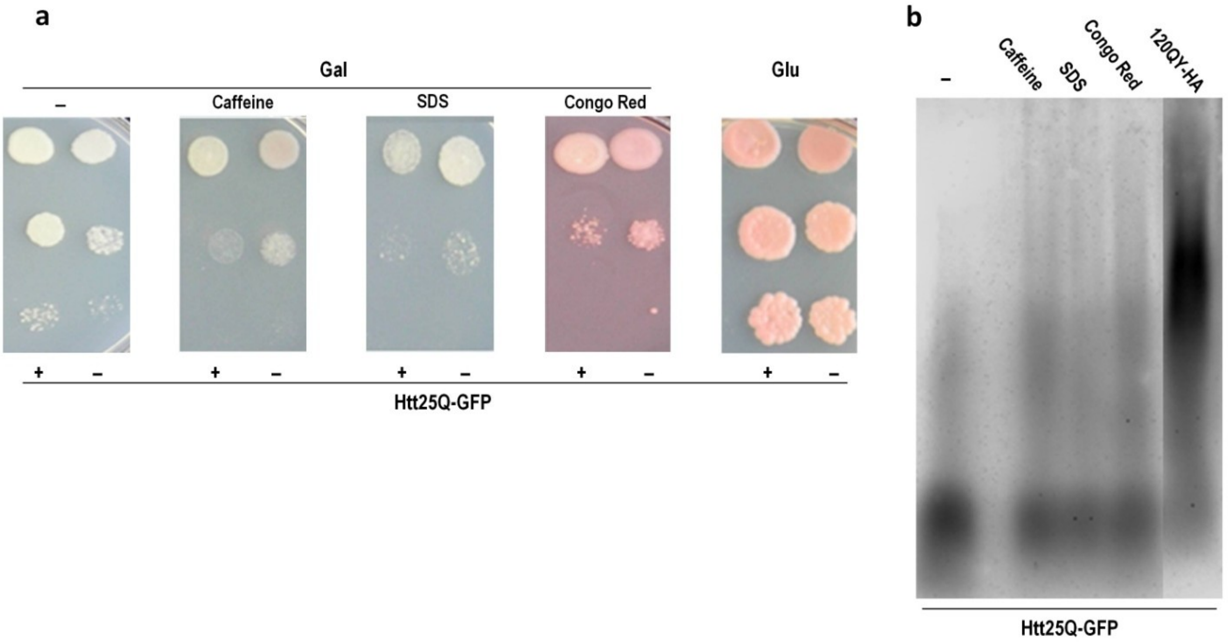

Figure S6 | Soluble Htt25Q-GFP is toxic for yeast in non-optimal growth conditions. (**a**) Growth of the 74-D694 [*psi*$^-$][*PIN*$^+$] transformants carrying either a centromeric plasmid expressing Htt25Q-GFP (+) or an empty vector (-) on media containing containing 0.006% SDS, 3 mM caffeine or 5 µg/ml Congo Red. Three serial 5-fold dilutions of cell suspensions are shown. (**b**) Htt25Q-GFP does not form polymers in cells grown in media containing SDS, caffeine or Congo Red. Polymers generated by Htt25Q-GFP upon co-production with 120QY-HA are shown for comparison. For other details, see legend to Fig. 1.
